# Mitochondria-associated endoplasmic reticulum membranes (MAMs): Possible therapeutic targets in heart failure

**DOI:** 10.3389/fcvm.2023.1083935

**Published:** 2023-01-26

**Authors:** Yu Zhang, Jiayu Yao, Mingming Zhang, Yushan Wang, Xingjuan Shi

**Affiliations:** Key Laboratory of Developmental Genes and Human Disease, School of Life Science and Technology, Southeast University, Nanjing, China

**Keywords:** mitochondria-associated membranes (MAMs), cardiovascular diseases, myocardial infarction, cardiomyopathy, heart failure

## Abstract

Mitochondria-associated endoplasmic reticulum membranes (MAMs) are formed by physical connections of the endoplasmic reticulum and mitochondria. Over the past decades, great breakthroughs have been made in the study of ER-mitochondria communications. It has been identified that MAM compartments are pivotal in regulating neurological function. Accumulating studies indicated that MAMs participate in the development of cardiovascular diseases. However, the specific role of MAMs in heart failure remains to be fully understood. In this article, we first summarize the structural and functional properties of MAM and MAM-associated proteins. We then focus on the roles of MAMs in myocardial infarction, cardiomyopathy and heart failure, and discuss the involvement of MAMs in disease progression and treatment. Elucidating these issues may provide important insights into therapeutic intervention of heart failure.

## Introduction

Mitochondria and endoplasmic reticulum (ER) are the two essential organelles which are tightly intertwined in eukaryotic cells (1). Mitochondria are the core parts of cell energy metabolism in maintaining the cellular function. Whereas ER, also known as sarcoplasmic reticulum (SR) in myocytes, participates in calcium storage, protein folding and processing, lipid metabolism (2). Mitochondria-associated ER membranes (MAMs), membranous contact sites between mitochondria and ER, bidirectionally regulates organelle physiological functions like lipid and Ca^2+^ homeostasis, mitochondrial dynamics, autophagy and apoptosis. Interruption of ER-mitochondria communication is a major cause of altered cellular homeostasis, which can lead to serious diseases including cancer, neurological diseases and cardiovascular diseases (CVDs) (3).

Cardiovascular diseases are the leading cause of death in the world, which consists of hypertension, acute myocardial infarction (AMI), cardiomyopathy, heart failure and other cardiac problems (4). Heart failure, the common end-stage of most cardiovascular diseases, is caused by hypertension, myocardial infarction (MI), ischemia and cardiomyopathies (5). In this article, we first describe the structural and functional properties of MAMs in cardiomyocytes, and then focus on their function in the development of heart failure. Interpretation of these issues may provide important diagnostic value and potential targets for heart failure.

### Structure and composition of MAMs

Mitochondria-associated ER membranes, composed of ER subdomains placed alongside with the outer membrane of mitochondria (OMM), can fluctuate dynamically. Electron microscopy revealed a distance of approximately 10–25 nm between the ER and OMM (6). The two organelles maintain stable and dynamic communications by the protein tethers. Proteomics evaluation demonstrated that MAMs components, highly conserved among different species and different tissues, play a direct physical tethering connection role or act as modulators of the tethering complexes in MAMs (7).

#### IP3Rs—GRP75-VDACs complex

The ER Ca^2+^ channel inositol 1,4,5-triphosphate receptors (IP3Rs) physically connect with OMM voltage-dependent anion channels (VDACs) *via* the cytoplasmic chaperone glucose-regulated protein 75 (GRP75), forming a tripartite complex to modulate ER-mitochondria juxtaposition (8). In mouse primary neurons, GRP75 promotes ER-mitochondria tethering and mitochondrial Ca^2+^, thus enhancing ATP production (9).

#### VAPB–PTPIP51 or ORP5/8 complex

The ER membrane protein vesicle-associated membrane protein associated protein B (VAPB), binds to the OMM protein tyrosine phosphatase-interacting protein-51 (PTPIP51), forming VAPB-PTPIP51 tethering complex which regulates ER-mitochondria Ca^2+^ transmission (10). Disruption of their interaction causes MAMs dissociation, and disturbs mitochondrial Ca^2+^ import and ATP synthesis (11). Besides, oxysterol-binding protein-related protein 5/8 (ORP5/8), enriched at MAMs in mammalian cells, physically interacts with PTPIP51. Inhibition of ORP5/ORP8 contributes to mitochondria morphology defects and respiratory dysfunction (12).

#### MFN2–MFN1/2 complex

Mitofusin2 (MFN2), mitochondrial fusion regulator, is recognized as an important constituent of MAMs. ER-resident MFN2 forms homodimer or heterodimer with either mitofusin (MFN1) or MFN2 on the OMM (13). MFN2 depletion promotes the ER-mitochondria connections and mitochondrial Ca^2+^ uptake from ER, indicating that MFN2 is more than a physical tether (14, 15).

#### BAP31-Fis1 complex

During the apoptotic process, the ER-located B-cell receptor-associated protein 31 (BAP31) is associated with OMM protein, the mitochondrial fission 1 protein (Fis1), acting as another tether for MAMs to induce apoptosis (16). Besides, phosphofurin acidic cluster sorting protein 2 (PACS-2), the first MAM protein identified to be involved in MAM formation, regulates the tethering of mitochondria with ER in a BAP31-dependent manner (17).

### Function of MAMs

**FIGURE 1:**
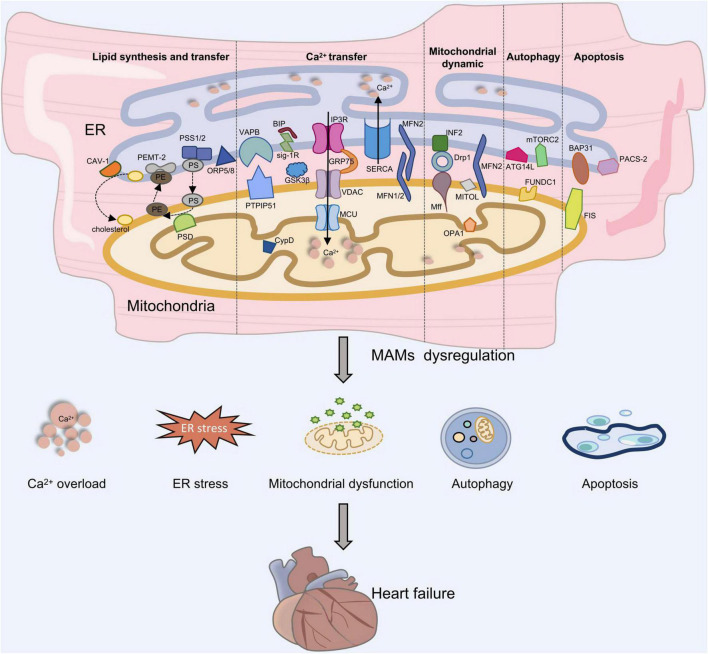
The role of MAMs in heart failure development. Proteins located on the ER surface, such as IP3R, VAPB, MFN2, and BAP31 interact with their counterparts on the OMM like VDAC, PTPIP51 or ORP5/8, MFN1/2, and Fis1, forming four major tethering complexes at MAMs in mammalian cells. Depletion of MAMs can lead to Ca^2+^ overload, ES stress, mitochondrial dysfunction, autophagy and apoptosis, which is involved in the development of heart failure.

Increasing evidence suggests that MAMs provide a platform for maintaining intracellular homeostasis and biological functions (18) (Figure 1).

#### Lipid synthesis and transfer

MAMs are abundant in proteins involved in lipid metabolisms including phosphatidylserine synthase (PSS), fatty acid CoA ligase 4 (FACL4), phosphatidylethanolamine N-methyltransferase 2 (PEMT2), phosphatidylserine decarboxylase (PSD) (19), as well as in phospholipid, triacylglycerol synthesis and steroidogenesis (20). Caveolin-1 (CAV-1) is an integral component distributed abundant on MAMs, which promotes lipid and cholesterol metabolism (21).

#### Ca^2+^ transfer and signal transmission

Ca^2+^ is a second messenger modulating multiple cellular activities such as cell metabolism and apoptosis (22). As mentioned above, the effective Ca^2+^ transmission at MAMs is regulated by multiple protein complexes. IP3R1-GRP75-VDAC1 tethering complex forms a Ca^2+^ regulatory axis with mitochondrial calcium uniporter (MCU), which mediates Ca^2+^ transmission from ER to mitochondria (23). In addition, ER chaperone proteins like Sigma-1 receptor (Sig-1R) physically associate at MAM, and regulate Ca^2+^ transmission through IP3R3 (24).

#### Mitochondrial dynamics

Mitochondrial dynamics include mitochondrial fission and fusion, which are crucial for maintaining cellular homeostasis. Proteins involved in mitochondrial dynamics are enriched in MAMs (25). MFN1 and MFN2 regulate OMM fusion, while Optic atrophy 1 (OPA1) modulates IMM fusion. The mitochondrial fission process is regulated by dynamin-related protein1 (Drp1), Fis1, mitochondrial fission factor (Mff), and mitochondrial dynamic proteins of 49 and 51 kDa (MiD49/51). A recent study emphasized that MAMs are the origin sites of mitochondrial fission (26).

#### Autophagy and apoptosis

Many autophagic proteins are located in MAMs and autophagosomal membranes may originate from MAMs (27). Under starvation, the pre-autophagosome marker autophagy-related 14-like (ATG14L) promotes relocalization of the autophagy induction factor mammalian target of rapamycin complex 2 (mTORC2) to MAMs and initiates autophagosome formation (28). It is reported that decreased MAMs tethering complexes result in abnormal hippocampal autophagy in rats (29). Moreover, MAMs modulate apoptosis *via* Ca^2+^ regulation. Evidence showed that Ca^2+^ overload can induce mitochondrial permeability transition pore (mPTP) opening and apoptosis (30). BAP31-Fis1 tethering complex recruits procaspase-8, which promotes the release of Ca^2+^ stores in ER and causes apoptosis (16).

### Role of MAM-associated proteins in CVDS

#### Ischemia-reperfusion injury

MI is an acute syndrome of CVD with high death rate (31). Myocardial ischemia/reperfusion (I/R) injury, a pathophysiological status after the ischemic myocardium returns to normal perfusion, is commonly deemed as a serious risk factor for coronary artery disease (32). MI is characterized by cardiac injury, myocardial cell death and abnormal cardiac function, which will lead to heart failure and death (33). Myocardial I/R injury is mainly associated with oxidative stress, ER stress and mitochondrial dysfunction (34). Among these, calcium overload is the leading cause of disordered oxidative phosphorylation and contributes to mitochondrial dysfunction (25).

Several components of ER/SR-mitochondria tethering complex participate in Ca^2+^ homeostasis and mediate mPTP opening and I/R damage. Cyclophilin D (CypD), encoded by *Ppif*, is located in the mitochondrial matrix and acts as a crucial regulator for mPTP opening and necrosis. CypD interacts synergistically with the VDAC1-Grp75-IP3R1 complex and enhances ER Ca^2+^ efflux into mitochondria (Table 1) (35). CypD overexpression induced mPTP opening without stimulating cell death, whereas CypD inactivation significantly reduced myocardial infarct size during I/R and ameliorated myocardial injury *via* impeding Ca^2+^ overload (36, 37). Notably, the protective effect of preconditioning was absent in *Ppif*^–/–^ mice, which were more susceptible to heart failure, indicating that CypD plays a dual function in I/R (38, 39).

**TABLE 1:** The roles of MAMs related proteins in CVDs.

Disease	Protein	Function in MAM	Model	Expression	Role in CVD	References
Ischemia-reperfusion injury	CypD	Ca^2+^ transmission	Mice	↑	CypD inactivation significantly ameliorates myocardial injury	(36, 37)
	GSK3β	Ca^2+^ regulatory	Mice Rabbit	↑	Inhibition of *GSK3*β diminished Ca^2+^ overload and reduced myocardial apoptosis	(40, 41)
	PTPIP51	Calcium homeostasis	Rat Mice	↑	Cardiac deletion of *PTPIP51* strikingly alleviates cardiac injury	(42, 43)
	SERCA	ER Ca^2+^ uptake pump	Mice	↑	Overexpression of SERCA protects microcirculation against cardiac I/R injury	(44, 45)
	MFN1/2	Calcium homeostasis	Rat Mice	↑	*Mfn1/2* deletion protects the heart against ischemia and reperfusion injury	(47, 48)
Cardiomyopathy	MFN2	Mitochondrial fusion	Mice	↑	*Mfn2*-deficient mice exhibits abnormal mitochondria, which induces respiratory dysfunction and causes DCM	(50, 51)
	Drp1	Mitochondrial fission	Mice	↑	Cardiac knockout of *Drp1* induces DCM with disregulated mitochondria	(53, 54)
	GSK3β	Calcium homeostasis	Mice	/	Cardiac deletion of *GSK-3* causes DCM and death	(49, 55)
	VDAC	Ca^2+^ channel on OMM	Mice	↑	Cardiac *VDAC2* knockout mice showed defected cardiac function and DCM	(56, 57)
Heart failure	IP3R	ER Ca^2+^ channel	Mice Rat Human	↑	Inhibition of IP3R1 alleviates myocardial injury and heart failure	(66, 67)
	FUNDC1	Regulate Ca^2+^ and autophagy	Mice Human	↓	*FUNDC1* deletion causes mitochondrial dysregulation, cardiac dysfunction and heart failure	(68, 69)
	SIG-1R	ER chaperon	Mice	↓	*Sig-1R* knockout mice demonstrate mitochondrial dysfunction and heart failure	(70, 71)
	MFN2	Ca^2+^ transmission	Rat Mice Human	↓	Cardiac deletion of *MFN2* mice developed cardiac hypertrophy and diastolic dysfunction	(74–76)
	Drp1	Mitochondrial dynamics and mitophagy	Mouse	↑	Cardiac deletion of *Drp1* showed progressive ventricular enlargement and functional decompensation, leading to heart failure	(79–81)
	OPA1	Mitochondrial dynamics	Rat Human	↓	Reduced OPA1 promoted apoptosis and mitochondria fragmentation, which causes heart failure progression	(83, 84)

Glycogen synthase kinase-3 beta (GSK-3β), a new Ca^2+^ regulator located in the SR/ER, specifically interacts with the IP3R Ca^2+^-channeling complex and regulates Ca^2+^ transfer in cardiomyocytes (40). GSK-3β inhibition diminished Ca^2+^ overload and reduced myocardial apoptosis resulted from I/R, thereby providing cardioprotection (40). Moreover, GSK-3β inhibitors attenuated infarct size in mice and rabbits, indicating drug administration was a feasible method (41). VAPB-PTPIP51 is a widely accepted tethering complex in MAMs. PTPIP51 is markedly increased in mice I/R hearts. PTPIP51 overexpression mediated excessive mitochondrial Ca^2+^ uptake, but reversed by MCU inhibition, which protected cardiomyocytes against PTPIP51-mediated apoptosis (42). Cardiac knockdown of *PTPIP51* strikingly alleviates cardiac injury after myocardial I/R, indicating that PTPIP51 might be a potential target for ischemic heart disease. Furthermore, downregulation of VAPB or PTPIP51 promotes autophagy by reducing mitochondrial Ca^2+^ levels (43).

Sarco/endoplasmic reticulum Ca^2+^-ATPase (SERCA), the main pump for Ca^2+^ uptake in the ER, regulates calcium homeostasis by interacting with calnexin. SERCA ameliorated reperfusion-induced myocardial injury by employing gene delivery strategies targeting SERCA (44, 45). Mitochondrial dynamics is crucial in myocardial I/R by regulating mPTP opening (46). MFN1-MFN2 complex is implicated in modulating mitochondrial fission and maintaining ER-mitochondria microdomain (13). Acute deletion of *Mfn1* and *Mfn2* prevented myocardial I/R injury and reduced infarct size (47). *Mfn2* knockout hearts exhibited resistance to I/R injury, however long-term *Mfn2* deletion contributed to cardiac dysfunction (47, 48).

#### Cardiomyopathy

Cardiomyopathy, myocardial disorder with abnormal cardiac muscle, can be either acquired or inherited. Hypertrophic cardiomyopathy (HCM) and dilated cardiomyopathy (DCM) are the most common cardiomyopathies. Among these, DCM is the most common cause of heart failure, which is characterized by structural thinning and dilation of heart chambers with a progressively defected cardiac function (49).

It has been reported that cardiac deficiency of *Mfn1* and *Mfn2* exhibits progressive DCM and heart failure in succession (50). Structural and functional abnormal mitochondria were observed in *Mfn2*-deficient mice, which induced respiratory dysfunction and caused DCM (51). Moreover, mice with Drp1 mutation demonstrated cardiomyopathy with punctuate calcification in the heart (52). Cardiac depletion of *Drp1* induced DCM after birth and rapid death in mice (53, 54). Increased mitochondrial connection, accumulated ubiquitinated proteins as well as reduced respiration was observed in *Drp1* knockout cardiomyocytes. These studies indicate that Drp1 is crucial in regulating mitochondrial quality and myocardial survival.

Adult cardiac deletion of a multifunctional regulator GSK-3 contributed to severe DCM due to cell cycle dysregulation, indicating that GSK-3 is involved in maintaining cardiac homeostasis (49, 55). VDAC, the most abundant mitochondrial outer membrane protein, contains three subtypes-VDAC 1, 2, and 3 in mammalian cells (56). It has been reported that VDAC1 is upregulated in the left ventricle of HCM patients (57). VDAC1 inhibition significantly attenuated mitochondrial Ca^2+^ overload and protected cells from hypoxia-reoxygenation (H/O) (58). Cardiac *VDAC2* knockout mice showed decreased ejection fraction and increased brain natriuretic peptide (BNP) level and cardiac fibrosis, which was consistent with DCM features (56).

#### Heart failure

Heart failure is a complicated pathophysiological syndrome of cardiac pumping failure (5). Conditions such as ischemia, pressure or volume overload, cardiac hypertrophy, cardiomyopathy, will eventually lead to heart failure (59). It is well recognized that mitochondrial function and Ca^2+^ homeostasis are significant in cardiac remodeling and heart failure (60–62). The cardiac rhythmicity and contraction require energy, which is driven by mitochondrial oxidative phosphorylation (63). Besides, SR, a membrane system with a high density of Ca^2+^-ATPases, maintains optimal calcium levels for myocardial contraction. Disruption of Ca^2+^ homeostasis can trigger ER stress and energy metabolism defects, affecting the development of heart failure (64, 65).

Mitochondrial Ca^2+^ dysregulation is involved in cardiac remodeling and heart failure. IP3R is a ligand-gated calcium channel located in the ER/SR, with isoform IP3R-2 predominant in the heart. IP3R is associated with cardiac remodeling in response to various stress that cause hypertrophy (66). The expression and activity of IP3R is enhanced under pathological conditions such as cardiac hypertrophy and heart failure. Moreover, inhibition of IP3R1 alleviates myocardial injury and heart failure (67). FUNDC1, a highly conserved OMM protein, maintains MAM formation by interacting with IP3R2 and regulates mitophagy (68). Compared with healthy group, the expression level of FUNDC1 and the number of SR-mitochondria contacts are dramatically reduced in heart failure patients. The decreased FUNDC1 level in MAMs contributed to impaired SR Ca^2+^ transportation to mitochondria through inhibition of IP3R2 ubiquitin-dependent degradation, resulting in perturbation of the CREB/Fis1 pathway and eventually compromising cardiac function. Besides, *FUNDC1* knockout mice showed diastolic and systolic dysfunction (69). In contrast, FUNDC1 overexpression elevates both cytosolic and mitochondrial Ca^2+^ levels in cardiomyocytes, and lowers SR Ca^2+^ levels. Sig-1R disassociated from the binding immunoglobulin protein (BiP) and prolonged mitochondria Ca^2+^ uptake *via* IP3R under ER stress (70). Sig-1R regulates Ca^2+^ transfer into mitochondria to promote ATP production (71). *Sig-1R* knockout mice displayed mitochondrial dysfunction and cardiac remodeling, causing cardiac dysfunction. Besides, Fluvoxamine, possessing high Sig-1R affinity, alleviated heart failure in both mice and rat models subjected to TAC (72).

Mitochondrial dynamics is participated in cardiac hypertrophy and heart failure progression. Cardiac depletion of *Mfn1/2* in mice showed impaired heart function with increased left ventricular end-diastolic volume and decreased fractional shortening (48, 73). Accumulating studies have demonstrated that MFN2 was downregulated in heart failure models induced by spontaneously hypertensive rats (SHR) or TAC (74). Consistently, MFN2 was decreased in hypertrophic cardiomyocytes induced by Angiotensin II (Ang II), accompanied by the alterations of mitochondria morphology (75). A study showed that *MFN1/MFN2* double knockout mice died at the embryonic stage due to heart failure (50). Besides, cardiac deletion of *MFN2* mice developed cardiac hypertrophy and moderate diastolic dysfunction (76). Conversely, MFN2 overexpression alleviated Ang-II induced cardiac hypertrophy (77). Intriguingly, sex hormones (estrogen and testosterone) can increase cardiac expression of Mfn1 and Mfn2, suggesting that further study is needed on the regulatory effect of the sex hormones, and their cardioprotective effects (78). Drp1, mitochondrial fission regulator, is upregulated in damaged cardiac tissues induced by doxorubicin. A study showed that Drp1 inhibitor will be a promising pharmacological agent, which inhibits the excessive mitochondrial fission mediated by doxorubicin and ameliorates its cardiotoxicity (79). Drp1 deficiency in adult mouse hearts showed the pathophysiological consequences of progressive ventricular enlargement and functional decompensation, resulting in heart failure (80). Drp1-dependent mitochondrial autophagy exerts a protective role in mitochondrial dysfunction and heart failure resulted from pressure overload (81). At present, microRNAs (miR) are being studied as therapeutic targets for CVDs. It is reported that miR 499 protects heart against MI by inhibiting mitochondrial fission mediated by Drp1 (82). OPA1, mediates IMM fusion and acts as a crucial regulator of morphological change in cardiac physiology. Altered OPA1 function was proposed to lead to the pathogenesis of heart failure (83). Studies have shown that protein levels of OPA1 were reduced in both rat and human heart failure models accompanied with mitochondrial fragmentation. Reduced OPA1 promoted apoptosis and mitochondria fragmentation, which may contribute to heart failure progression with progressive loss of cardiac myocytes (84). Together, mitochondrial dynamics are essential to maintain cardiac structure and function, which may act as a potential strategy to prevent myocardial hypertrophy and heart failure.

## Conclusion

MAMs, membranous contact sites between mitochondria and ER, regulate various cellular processes including Ca^2+^ homeostasis, mitochondrial dynamics, autophagy and apoptosis. Heart failure is the final stage of diverse CVDs. Accumulating studies have defined the essential function of MAMs in the development of heart failure. For instance, depletion of CypD contributed to reduced myocardial infarction and ameliorated cardiac function (36). Upregulation of PTPIP51 caused cardiac injury by promoting mitochondrial Ca^2+^ overload and apoptosis, whereas PTPIP51 depletion significantly protected the heart from I/R injury (42). Besides, *Mfn2* knockout mice developed dilated cardiomyopathy (51). Dysregulation of Drp1 in cardiomyocytes contributes to myocardial injury and heart failure (82). These studies indicate that MAMs may act as biomarkers and potential therapeutic targets in heart failure. The function of MAMs in cardiovascular diseases worth more attention due to their multifunctional. The improved understanding of MAMs integrity regulation or MAMs targets identification might provide significant therapeutic strategies for cardiovascular diseases.

## Author contributions

XS: conceived and designed the review. YZ, JY, MZ, and YW: collected the literatures. YZ, JY, and XS: wrote the manuscript. XS, MZ, and YW: reviewed and edited the manuscript. All authors contributed to the article and approved the submitted version.
